# Prevalence and geographical variation of Factor V Leiden in patients with cerebral venous thrombosis: A meta-analysis

**DOI:** 10.1371/journal.pone.0203309

**Published:** 2018-08-29

**Authors:** Xinyuan Li, Li Cui, Yunbo Li, Lijun Zhu, Chenglin Wang, Jing Liu, Shaokuan Fang

**Affiliations:** 1 Department of Neurology, Neuroscience Centre, the First Teaching Hospital of Jilin University, Changchun, China; 2 Department of Neurosurgery, the First Teaching Hospital of Jilin University, Changchun, China; 3 China-Japan Union Hospital of Jilin University, Changchun, China; Universitatsklinikum Freiburg, GERMANY

## Abstract

**Background:**

Previous results regarding the prevalence of Factor V Leiden (FVL) in patients with cerebral venous thrombosis (CVT) varied remarkably. Therefore, we performed a meta-analysis to evaluate the potential association between FVL and CVT.

**Methods:**

The PubMed, Embase, Cochrane Central Register of Controlled Trials, and Web of Science were searched for relevant case-control studies. The data were pooled for analysis of the association between FVL and CVT with a random-effects model. Subgroup and sensitivity analyses were performed to evaluate the robustness of the results. Publication bias was assessed by funnel plot and the Egger’s test.

**Results:**

A total of 39 case-control studies including 1807 cases of CVT and 7699 control cases were included. FVL was more common in patients with CVT (195/1822) than in controls(369/7795), [odds ratio(OR) = 2.70, 95% confidence interval(CI) 2.16–3.38, P < 0.00001]. Results of subgroup analyses indicated that the association between FVL and CVT varied according to geographic regions. The associations between FVL and CVT were significant in studies from Germany (OR = 2.42, 95%CI 1.70–3.45, P < 0.00001), Brazil(OR = 2.82, 95%CI 1.24–6.42, P = 0.01), France (OR = 5.44, 95%CI 1.35–21.92, P = 0.02), Iran (OR = 6.61, 95%CI 1.83–23.93, P = 0.004), and Tunisia (OR = 6.54, 95%CI 2.89–14.82, P < 0.00001), but not in those from Italy (OR = 1.49, 95%CI 0.59–3.78, P = 0.40) or the US (OR = 2.37, 95%CI 0.59–9.42, P = 0.22).

**Conclusion:**

FVL may be more common in patients with CVT. However, the association between FVL and CVT varied depending on the geographic origin of the studies.

## Introduction

Cerebral venous thrombosis (CVT) has been recognized as a rare disease that is primarily defined as thrombosis in cerebral veins, with a reported annual incidence of 3 to 4 cases per 2 million in adults and 7 cases per 1 million in neonates[1]. Clinically, CVT has been suggested to be a rare cerebrovascular disease that accounts for 0.5% of all cerebral strokes[2]. With the development of novel sensitive imaging techniques and increasing awareness of clinicians for the disease, the prevalence of CVT, as reported in recent studies, may be higher than previously estimated[3]. Although the exact cause of CVT remains to be determined, some risk factors for the disease have been proposed, such as surgery, trauma, immobilization, oral contraceptive pills, pregnancy, puerperium, and others. Moreover, recent evidence suggests that genetic factors related to thrombosis, such as Factor V Leiden (FVL), prothrombin G20210A[4], and methylenetetrahydrofolate reductase variant C677T may also be involved in the pathogenesis of CVT.

Accumulating evidence suggests that FVL is an important genetic determinant of venous thromboembolism(VTE)[5]. Similarly, some studies have indicated FVL plays a considerable role in the occurrence of CVT. However, the results of these studies varied significantly according to different geographical regions. In previously published meta-analyses, Marjot et al[6] included 767 CVT cases and 4020 controls and concluded that FVL was significantly associated with CVT. This was further confirmed in a similar study by Lauw et al[7], which included 919 CVT cases and 3168 healthy controls. However, a few case-control studies have been published since these meta-analyses, and whether geographical factors contribute to the heterogeneity regarding the association between FVL and CVT remains to be determined. Therefore, in this updated meta-analysis, we aimed to evaluate the overall association between FVL and CVT and also explored whether this association differed according to the geographical origin of the studies.

## Methods

### Search strategy

We performed this meta-analysis according to the guidelines of the Preferred Reporting Items for Systematic Review and Meta-Analysis (PRISMA) working group (S1 File)[8] and Meta-analysis on Genetic Association Studies(S1 Table). We systematically searched electronic databases including PubMed, Web of Science, Embase, and Cochrane Center Register of Controlled Trials (CENTRAL) hosted by the Cochrane Library for relevant studies, and the last search was performed on Nov 10, 2017. No restrictions of language or the region of the study were applied. The search terms were (Factor V Leiden OR Factor V OR 1691A OR 1691) AND (cerebral venous thrombosis OR cerebral vein thrombosis OR cerebral sinus thrombosis OR intracranial venous thrombosis OR intracranial vein thrombosis OR intracranial sinus thrombosis). The detailed search strategy was available (S2 File). The search was supplemented by comprehensive manual search of the references of the related studies or reviews, conference abstracts, letters to editor, or key review articles.

### Inclusion and exclusion criteria

Studies were included if they met the following eligibility criteria: (1) case-control studies; (2) enrollment of patients with cerebral venous or sinus or cortical vein thrombosis, which was objectively confirmed by accepted imaging methods (computed tomography, magnetic resonance imaging, magnetic resonance venography, magnetic resonance black-blood thrombus imaging, or angiography) or objectively documented by medical records; (3) control participants were non-genetically related subjects without a history of venous thromboembolism; (4) both cases and controls underwent genetic testing for FVL; and (5) the frequencies of FVG1691A for both CVT cases and controls were reported in original studies or could be calculated.

Studies were excluded from the current meta-analysis if they met the following criteria: (1) CVT was neither confirmed by imaging methods or documented by medical files; (2) genetic testing for FVL was not performed for the control group; or (3) data of interest were not available. If two studies with considerable overlapped (>50%) population were retrieved, the one most recently published study or the study of highest quality was included to avoid publication bias[7].

### Data extraction

Two authors (Xinyuan Li and Li Cui) independently assessed the eligible studies and extracted data using standard abstraction forms. Disagreements were resolved by reaching a consensus with a third author. In addition, we attempted to contact the corresponding author to request missing data via E-mail. The following information for each study was collected: the first author’s name, year of publication, number of participants, country, study design, age, sex, diagnostic methods of CVT, source of control, and FVL genotype frequencies in case and control groups.

### Quality assessment

Two authors (Xinyuan Li and Yunbo Li) independently evaluated the quality of the included studies according to the Newcastle-Ottawa Scale (NOS) for observational studies[9], and disagreements were resolved by reaching a consensus with a third author(Shaokuan Fang). The NOS consists of three main parts: selection (4 stars), comparability (2 stars), and outcome (3 stars) and ranges from 0–9 stars[10]. Thus, the quality of each study was determined on a scale from 0–9 points. Studies with 7–9 points were regarded as “high quality”, with 4–6 points as “moderate quality”, and with less than 4 points as “low quality”[11].

### Statistical analysis

The primary outcome of the present study was defined as the odds ratio(OR) for the prevalence of FVL in patients with CVT as compared with that in healthy controls. We used the Review Manager 5.3 (Cochrane Collaboration, London, UK) and Stata (Version 12.0) software for statistical analyses. The significance of the pooled estimates was determined by Z statistic with 95% confidence interval (CI), and statistical significance was set at a two-tailed P<0.05. Data were pooled when there were at least three case-control studies. ORs were combined using the Mantel-Haenszel method and a random-effects model that takes into account the variability among the included studies. Study heterogeneity was evaluated by the I^2^ statistic: a value of<50% indicated low to moderate heterogeneity, ≥50% indicated substantial heterogeneity. In general, significant heterogeneity was defined as I^2^≥50% and P<0.05. The genotypes of FVL mutation (G1691A) consisted of normal homozygote (G/G), mutated homozygote (A/A), and heterozygote (G/A). We regarded homozygote (A/A) and heterozygote (G/A) carriers of FVL as one group due to the rarity of homozygote carriers (A/A). Thus, a dominant model was applied. We performed a subgroup analysis based on geographical regions of the studies to identify whether this factor contributed to the heterogeneity among the included studies. We were unable to conduct other subgroup analyses (race/ethnicity, education level, family income, and disease severity) owing to studies not providing sufficient stratified data. We used a sensitivity analysis to evaluate the robustness of the results by omitting one study at a time. The publication bias was assessed by the funnel plot and the Egger’s test [12–13].

## Results

### Database search

**Fig 1:**
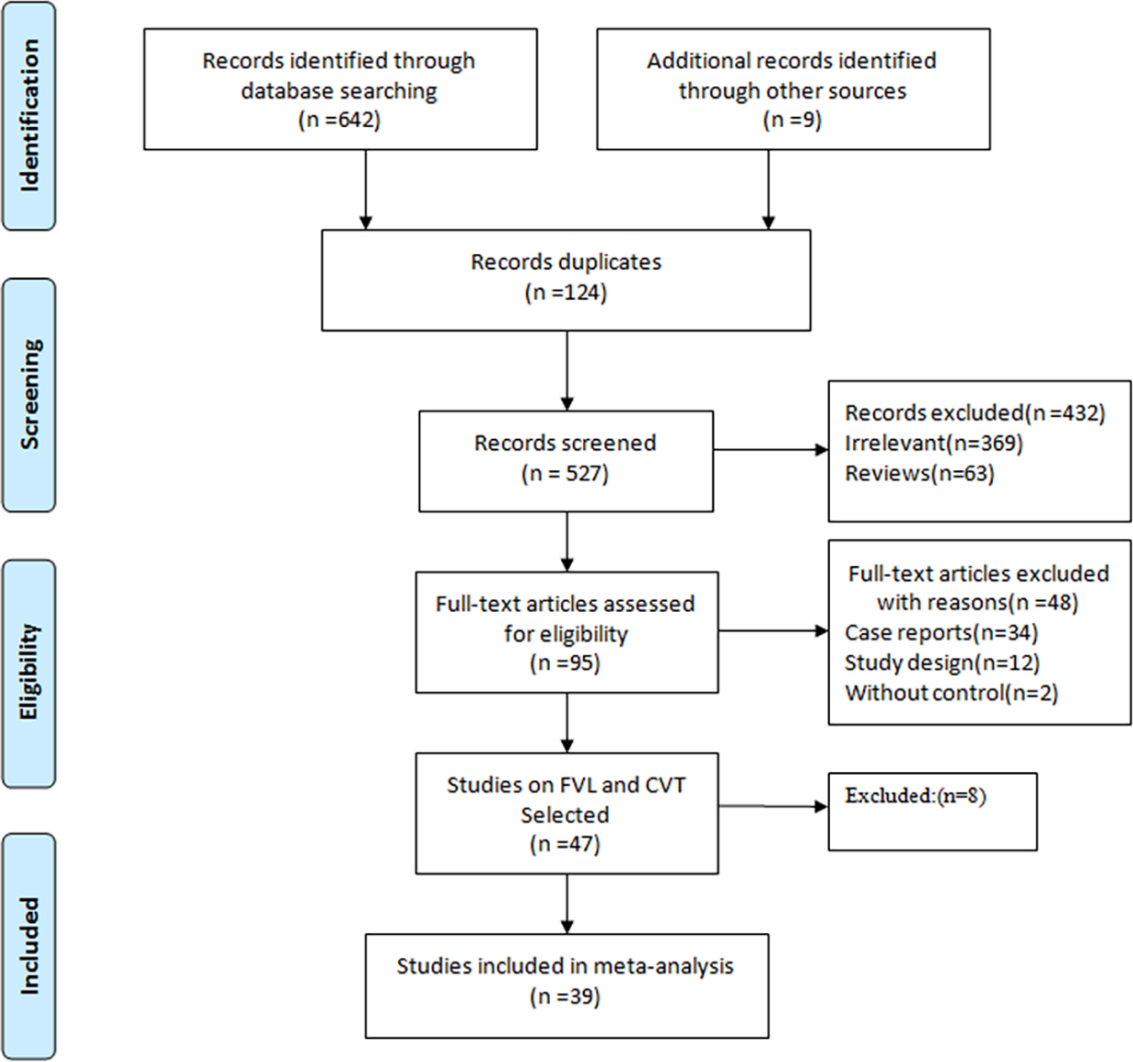
PRISMA flow diagram. FVL, Factor V Leiden; CVT, cerebral venous thrombosis.

A total of 651 articles were initially retrieved from the electronic database search (290 from PubMed, 240 from Web of Science, 86 from Embase, and 26 from CENTRAL). Then 369 irrelevant articles, 124 duplicate papers, and 63 reviews were excluded based on screening of the title and abstracts. Among the remaining 47 studies, characteristics of the eight studies[14–21] excluded from analysis were presented(S2 Table). Seven were excluded due to the overlapped population of already included studies, and the other one was excluded because no FVL was detected in cases or controls. Thus, 39 studies(S3 File) were included in the current meta-analysis. Fig 1 illustrated the flowchart of database search and study identification and Table 1 summarized the characteristics of the included studies.

**Table 1:** Characteristics of the included studies.

Study	Year	Country	CVT ascertainment	CVT(N)	Male/ female (N)	Mean or median age(range), years	Controls (N)	Male/ female (N)	Age	Matched
**Zuber**	1996	France	MRI and/or angiography	19	4/15	- (20–72)	57	-	-	Age
**Weih**	1998	Germany	MRA or angiography	12	2/10	33.8 (21–60)	36	6/30	34.2 (-)	Age
**Junker**	1998	Germany	MRA or DSA	58	20/38	32.5 (0.3–73)	105	50/55	44.0 (10–55)	Ethnical background
**Hillier**	1998	UK	CT,MRI,MRV or autopsy	15	2/13	35.3 (17–76)	300	-	-	-
**Lüdemann**	1998	Germany	MRI and/or DSA	55	15/40	40.0 (11–83)	272	-	- (18–55)	Same region
**Hagstrom**	1998	US	MRI and/or angiography	9	-	-	65	-	-	-
**Schobess**	1999	Germany	CT, MRI and venography	15	-	-	100	-	-	Age and sex
**Madonna**	2000	Italy	MRI and/or Angiography	10	4/6	35.1(-)	259	115/144	36.7	Age,sex and ethnic background
**Voetsch**	2000	Brazil	CT,MRI and/or angiography	14	4/10	24 8 (16–31)	225	99/126	34.1 (16–50)	Age,sex,ethnic background
**Margaglione**	2001	Italy	Documented objectively.	28	13/15	- (-)	1304	576/743	36.0 (22–66)	-
**Bombeli**	2002	Switzerland	CT,MRI,venography or angiography	51	14/37	36.7 (17–61)	120	48/72	37.4 (19–62)	-
**Meng**	2002	China	Angiography	20	8/12	31.0 (20–48)	50	28/22	38.0 (18–58)	-
**Martinelli**	2003	Italy	CT,MRI and/or angiography	121	30/91	33.0 (12–64)	242	60/182	36.0 (13–62)	Age and sex
**Heller**	2003	Germany	CT,MRI,MRV or MRA	149	84/65	6.0 (0–18)	149	89/60	6.2 (0–18)	Age and sex
**Bonduel**	2003	Argentina	CT,MRI and/or MRA	23	20/3	- (0.2–16)	102	60/42	7.1 (0.2–15.9)	Age
**Rodrigues**	2004	Brazil	MRI and/or angiography	42	14/28	28.0(-)	134	54/80	34.0(-)	-
**Gadelha**	2004	Brazil	MRA or angiography	26	5/21	28.5 (3–46)	217	83/134	29.0 (15–62)	Age,sex and racial background
**Boncoraglio**	2004	Italy	CT,MRI,MRA or intra-arterial angiography	26	7/19	43.0 (21–73)	100	49/51	41.5 (21–72)	Ethnic background
**Ventura**	2004	Italy	CT or angiography	30	16/14	35.0 (16–49)	40	22/18	34.0 (18–51)	Age,sex and ethnic background
**Kenet**	2004	Israel	Documented objectively and confirmed by a neurologist	38	23/15	5.6(-)	112	60/52	6.3(-)	Age,gender distribution and ethnic origin
**Lichy**	2005	Germany	MRI and/or angiography	77	17/60	38.0(-)	203	85/118	36.5(-)	Same region
**Dindagur**	2006	India	MRI/MRV	86	0/86	23.5 (18–36)	86	0/86	24.0(-)	Age
**Miller**	2006	US	CT and/or MRI	24	-	-	437	-	-	-
**Stolz**	2007	Germany	MRI,MRA,CTA or DSA	121	28/93	43.5 (18–85)	120	30/90	43.4 (22–82)	Age,sex and same institution
**Colaizzo**	2007	Italy	Spiral CT or MRI	45	14/31	40.0 (13–55)	286	119/163	44.0(21–73)	Age,sex and social status
**Romero**	2007	Spain	CT and/or MRI	15	5/10	33.4(-)	60	-	-	Age and sex
**Altinisik**	2008	Turkey	Documented objectively	31	-	-	25	10/15	32.4 (22–47)	-
**Le Cam-Duchez**	2008	France	CT,MRI and/or angiography	54	11/43	37.1(-)	100	39/61	41.3(-)	-
**Koopman**	2009	Netherlands	Digital angiography, CT,MRI or surgery	19	4/15	36.0 (18–55)	19	-	-	Age and sex
**Rahimi**	2010	Iran	MRI	24	7/17	37.1(-)	100	50/50	36.1(-)	Age,sex and ethnic background
**Laugesaar**	2010	Estonia	MRV or CTV	7	-	-	400	207/193	-	-
**Cesarman-Maus**	2011	Mexico	MRI and/or angiography	40	7/33	28.2 (14–61)	145	0/145	36.7 (22–63)	-
**Ringelstein**	2012	Germany	MRI and/or angiography	136	33/102	41.0 (16–85)	1054	-	-(25–74)	Ethnic background
**Ben Salem-Berrabah**	2012	Tunisia	CT,MRI,MRV,MRA or autopsy	26	5/21	38.26 (15–72)	197	126/71	31.0 (14–54)	-
**Ashjazadeh**	2012	Iran	MRI/MRV	57	19/38	33.7(-)	50	-	-	Age,sex,geographical and ethnic background
**Orikaza**	2013	Brazil	CT,MRI or angiography	72	16/56	32.5 (8–69)	143	-	37.0 (18–66)	Age and sex
**Klai**	2013	Tunisia	MRI	51	0/51	30.0(-)	100	0/100	30.6(-)	Same geographic and socioeconomic group
**Tufano**	2014	Italy	Documented objectively	56	15/41	34.98(-)	184	50/134	35.05	Age and sex
**Saadatnia**	2015	Iran	MRI, venography or angiography	40	-	33.45(-)	51	-	30.75	Age and race
**Beye**	2017	Germany	CT,MRI and/or DSA	101	24/77	38.8 (18–76)	101	24/77	39.2 (19–70)	Age and sex

CVT, cerebral venous thrombosis; CT, computed tomography; MRI, magnetic resonance imaging; MRA, magnetic resonance angiography; MRV, magnetic resonance venography; DSA, digital subtraction angiography; CTA, computed tomography angiography; CTV, computed tomography venography;—,not applicable.

### Study characteristics

Overall, 39 case-control studies were included in the current analysis, with 1822 cases of CVT and 7795 controls. The included studies were published between 1996 and 2017. Among 39 studies, 14 studies did not report specific genotypes of FVL, 4 studies reported all genotypes including homozygous G1691A polymorphism, and the remaining 21 studies only reported heterozygous polymorphism. Thus, we considered homozygote (A/A) and heterozygote (G/A) carriers of FVL as one group using a dominant model. Additionally, we failed to detect the Hardy-Weinberg equilibrium(HWE) of controls.

### Quality assessment

The quality of included 39 case-control studies assessed by NOS was shown in Table 2. The scores from 4–9 were regarded as moderate and high quality.

**Table 2:** Details of quality assessment of the included studies.

Study	Year	Is the case definition adequate?	Representativeness of the cases	Selection of controls	Definition of controls	Comparability of cases and controls on the basis of the design or analysis		Ascertainment of exposure	Same method of ascertainment for cases and controls	Non- Response rate	Score
**Zuber**	1996	★	★	★	★	★		★	★	★	8
**Weih**	1998	★	★		★	★		★	★	★	7
**Junker**	1998	★		★				★	★	★	5
**Hillier**	1998	★	★	★	★			★	★		6
**Lüdemann**	1998	★	★	★				★	★	★	6
**Hagstrom**	1998	★	★					★	★	★	5
**Schobess**	1999	★	★			★			★	★	5
**Madonna**	2000	★	★	★	★	★		★	★	★	8
**Voetsch**	2000	★	★	★	★	★		★	★	★	8
**Margaglione**	2001		★	★	★			★	★	★	6
**Bombeli**	2002	★	★	★	★			★	★	★	7
**Martinelli**	2003	★	★	★	★	★	★	★	★	★	9
**Heller**	2003	★	★	★		★	★	★	★	★	8
**Bonduel**	2003	★	★	★				★	★	★	6
**Rodrigues**	2004	★	★	★	★				★	★	6
**Gadelha**	2004	★	★	★	★	★		★	★	★	8
**Boncoraglio**	2004	★	★					★	★	★	5
**Ventura**	2004	★		★		★		★	★	★	6
**Kenet**	2004	★	★	★				★	★	★	6
**Lichy**	2005	★	★	★	★			★	★		6
**Dindagur**	2006	★	★	★	★	★		★	★	★	8
**Miller**	2006	★	★	★				★	★		5
**Stolz**	2007	★	★	★		★		★	★	★	7
**Calaizzo**	2007	★	★	★	★	★	★	★	★	★	9
**Romero**	2007	★	★	★	★	★		★	★	★	8
**Altinisik**	2008		★					★	★	★	4
**Le Cam-Duchez**	2008	★	★	★	★	★		★	★	★	8
**Koopman**	2009	★	★			★		★	★	★	6
**Rahimi**	2010	★	★			★	★	★	★	★	7
**Laugesaar**	2010	★	★	★				★	★	★	6
**Cesarman-Maus**	2011	★	★		★			★	★	★	6
**Ringelstein**	2012	★	★	★	★			★	★	★	7
**Ben Salem-Berrabah**	2012	★	★					★	★	★	5
**Ashjazadeh**	2012	★	★	★	★	★		★	★	★	8
**Orikaza**	2013	★	★	★	★	★		★	★	★	8
**Klai**	2013	★	★	★	★			★	★	★	7
**Tufano**	2014		★	★	★	★		★	★	★	7
**Saadatnia**	2015	★	★			★		★	★	★	6
**Beye**	2017	★	★	★		★		★	★	★	7

★ evaluates the quality of included studies according to every part of NOS.

### Prevalence of FVL in CVT patients versus controls

**Fig 2:**
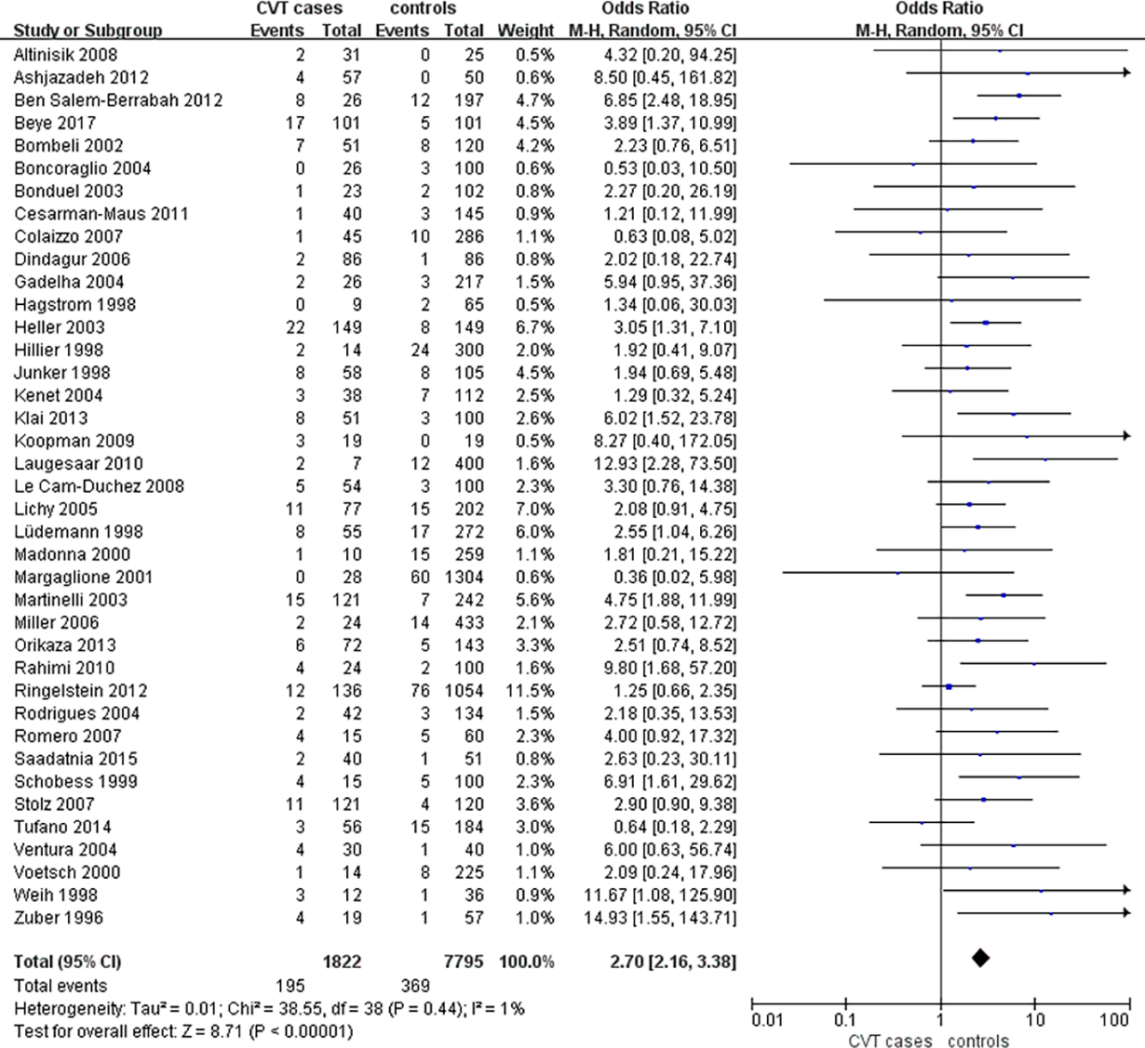
Forest plot of overall prevalence of FVL in CVT patients as compared with controls. CI, confidence interval; CVT, cerebral venous thrombosis.

Upon combining the data from all available studies, FVL/G1691A was found in 195/1822 CVT cases and 369/7795 controls (OR = 2.70, 95%CI 2.16–3.38, P<0.00001) (Fig 2).

**Fig 3:**
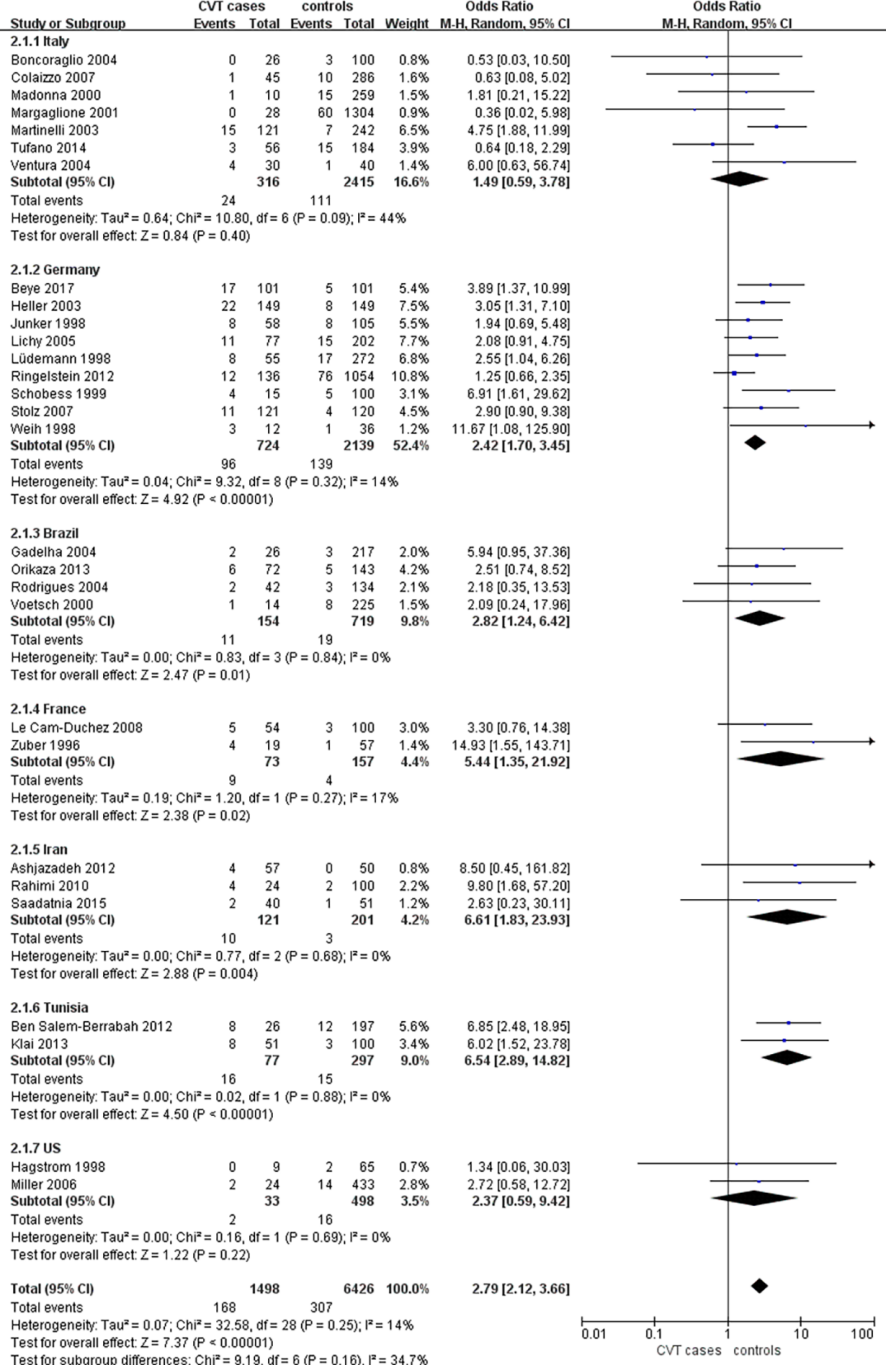
Subgroup analysis based on different countries. CI, confidence interval; CVT, cerebral venous thrombosis.

Subsequently, we performed a subgroup analysis based on region of the studies. The studies were performed in countries including Italy (7 studies), Germany (9 studies), France (2 studies), Brazil (4 studies), Iran (3 studies), Tunisia (2 studies), the US (2 studies), the UK (1 study), Switzerland (1 study), Argentina (1 study), Israel (1 study), India (1 study), Spain (1 study), Turkey (1 study), the Netherlands (1 study), Estonia (1 study), and Mexico (1 study). We performed subgroup analysis according to countries where the at least two studies were performed (Fig 3). Briefly, in Italy, FVL was found in 24/316 CVT cases and 111/2415 controls, and the prevalence was not significantly different (OR = 1.49, 95%CI 0.59–3.78, P = 0.40, I^2^ = 44%). In Germany, FVL was found in 96/724 CVT cases and 139/2139 controls(OR = 2.42, 95%CI 1.70–3.45, P<0.00001, I^2^ = 14%). In Brazil, FVL was found in 11/154 CVT cases and 19/719 controls (OR = 2.82, 95%CI 1.24–6.42, P = 0.01, I^2^ = 0%). In France, FVL was found in 9/73 CVT cases and 4/157 controls (OR = 5.44, 95%CI 1.35–21.92, P = 0.02, I^2^ = 17%). In Iran, FVL was found in 10/121 CVT cases and 3/201 controls (OR = 6.61, 95%CI 1.83–23.93, P = 0.004, I^2^ = 0%). In the two studies from Tunisia, the prevalence of FVL was higher in CVT cases than in controls (OR = 6.54, 95%CI 2.89–14.82, P<0.00001, I^2^ = 0%). In the US, the prevalence of FVL did not significantly differ between the groups (OR = 2.37, 95%CI 0.59–9.42, P = 0.22, I^2^ = 0%).

**Fig 4:**
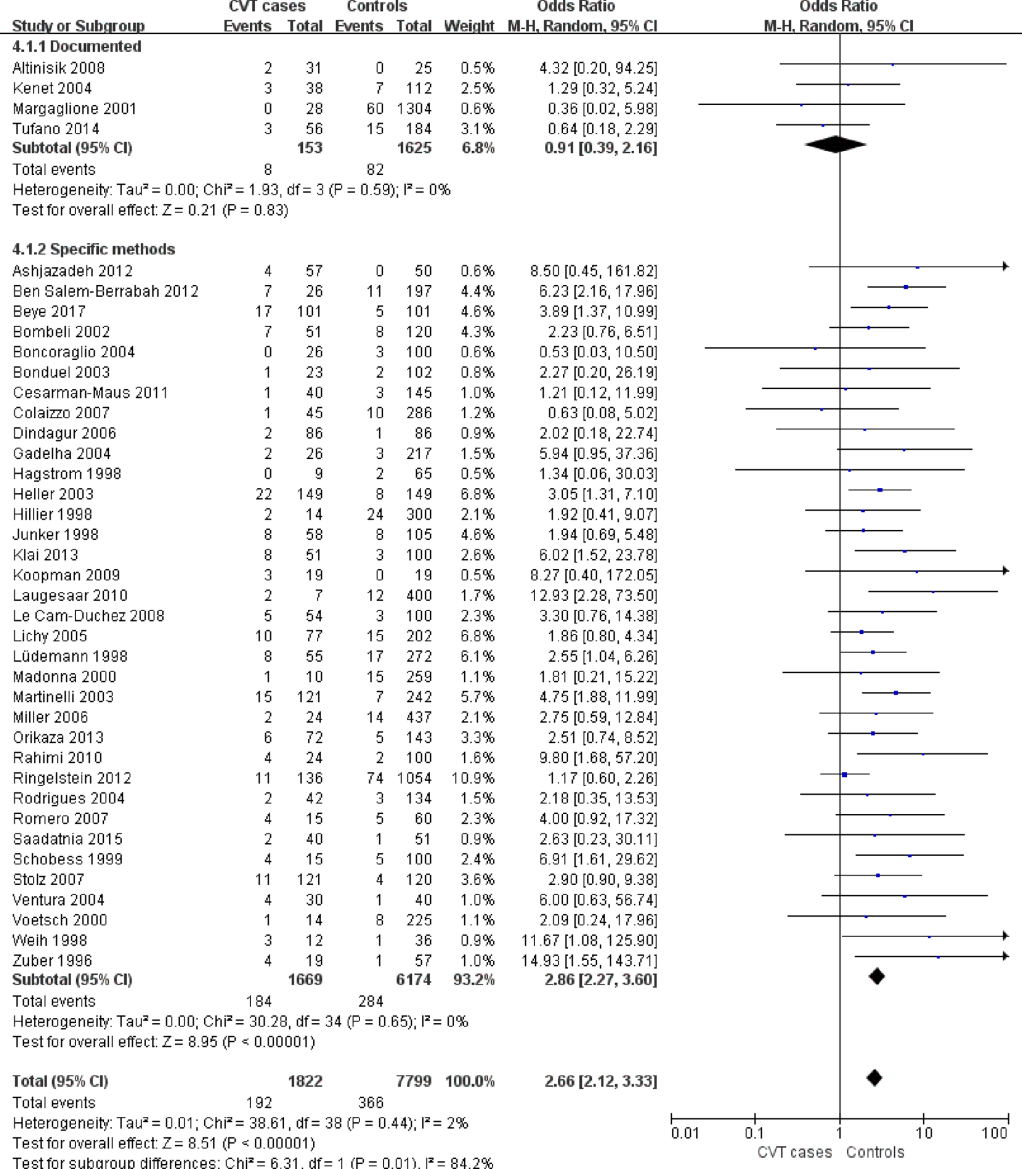
Subgroup analysis based on CVT ascertainment. CVT,cerebral venous thrombosis.

We also performed a subgroup analysis based on CVT ascertainment (Fig 4) to find the source of heterogeneity and found that the association was not significant between FVL and documented CVT (OR = 0.91, 95%CI 0.39–2.16, P = 0.83) and the prevalence of FVL was higher in cases of CVT diagnosed by specific radiological methods than in healthy controls (OR = 2.86, 95%CI 2.27–3.60, P<0.00001).

### Publication bias

**Fig 5:**
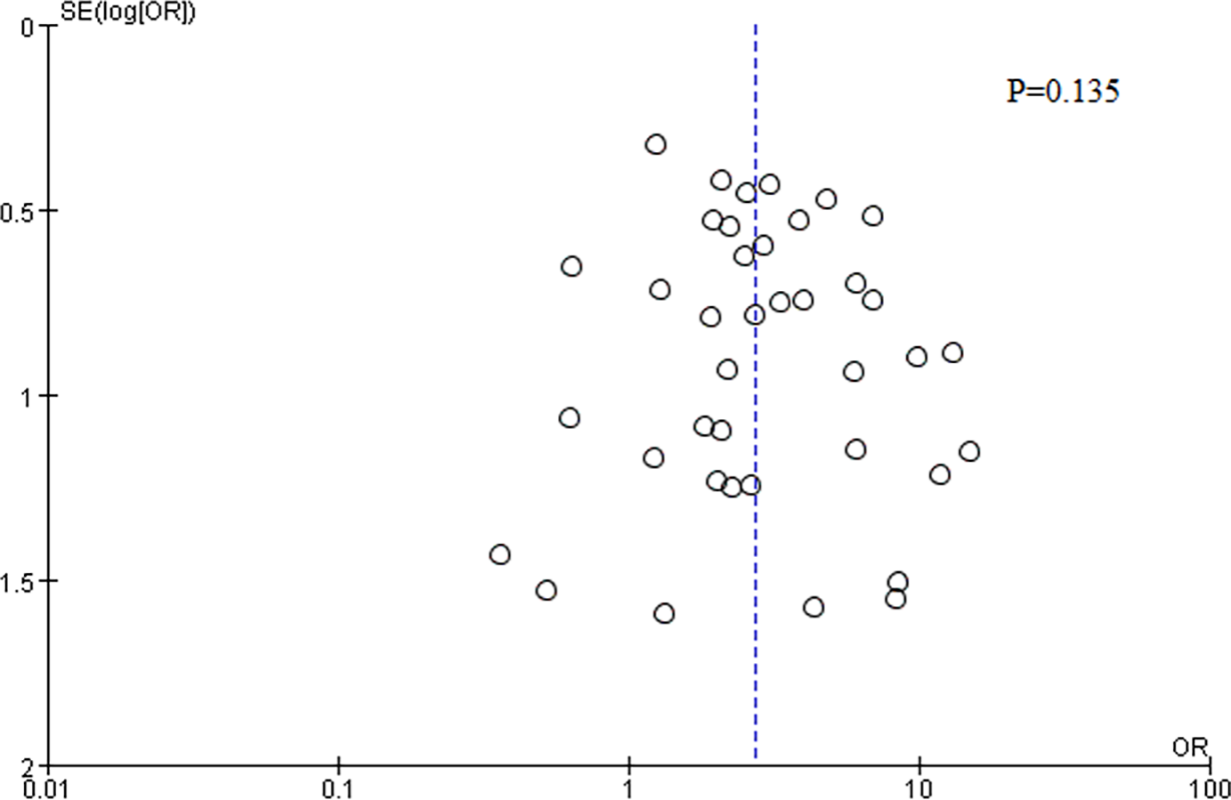
Funnel plot of publication bias.

There was no possibility of publication bias as indicated by the funnel plot and results of Egger’s test (P = 0.135) (Fig 5).

### Sensitivity analysis

**Fig 6:**
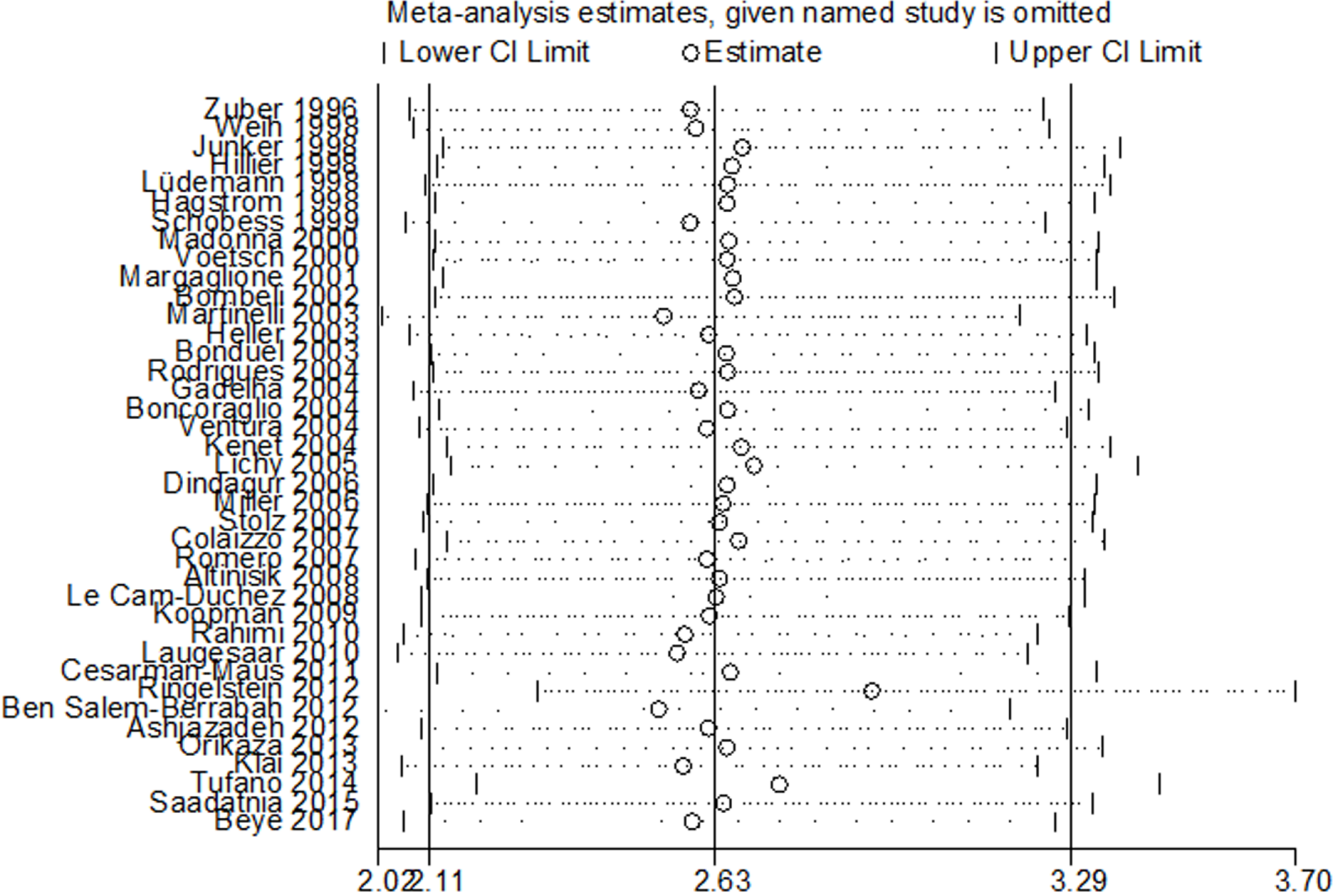
Sensitivity analysis of the overall prevalence of FVL. FVL, Factor V Leiden; CVT, cerebral venous thrombosis.

The results of sensitivity analysis for the prevalence of FVL indicated that the results were not significantly affected by excluding any one of the studies (Fig 6), suggesting the robustness of the results.

## Discussion

Although FVL has been established as the most common inherited risk factor for VTE[5,22], it has been confirmed that the prevalence of FVL in patients with deep vein thrombosis (DVT) and pulmonary embolism varies significantly among different regions and ethnicities[23–26]. However, whether the association between FVL and CVT is similar remains to be determined. To assess this issue, we performed a meta-analysis of 39 case-control studies including 1822 cases of CVT and 7795 controls and found that FVL was more common in patients with CVT than in health controls. However, the association between FVL and CVT varied depending on the geographic origin of the studies. To the best of our knowledge, this is the largest comprehensive meta-analysis for the overall prevalence of FVL mutation in patients with CVT. This is also the first evaluation of geographical variation of FVL in patients with CVT.

In general, we found that FVL was significantly associated with CVT, which was consistent with the results of previous reviews[6–7]. The current meta-analysis confirmed an overall significant association between FVL and CVT, but this association was highly dependent on the geography of patients. The association was significant between FVL and CVT in studies conducted in Germany, Brazil, France, Iran, and Tunisia, but not in those from Italy or the US. These results were consistent with previous reports that FVL is a contributory risk factor for the development of VTE[27–28]. However, it should be noted that the association between FVL and CVT was not statistically significant in Italy, which was inconsistent with the previous literature suggesting the prevalence of FVL in VTE was high in the Italian population[28]. Therefore, the potential association between FVL and CVT in studies from the above countries should be confirmed in future studies. Moreover, although the results of subgroup analyses were statistically insignificant in some studies, we found the results of subgroup analyses were generally similar to the overall prevalence of FVL. In addition, the results of subgroup analysis based on the CVT ascertainment showed that the prevalence of FVL was higher in studies in which CVT was diagnosed by specific radiological methods, but not in studies that included documented CVT. These analyses further confirmed the robustness of the results.

Our analysis had some strength as compared to previous reviews. In order to make our findings more robustness and credible, we applied several methods. First, in addition to electronic databases searches, we also conducted comprehensive manual-search of the reference lists of relevant reviews and tried our best to include all eligible studies. To date, our meta-analysis has the largest sample size to confirm the prevalence of FVL in CVT. In addition, this is also the first meta-analysis for geographical variation of FVL in patients with CVT and suggests that the prevalence of FVL varies from country to country. Second, all the included studies were of moderate to high quality as evaluated by NOS, which made the extracted data more reliable. Third, we performed sensitivity and subgroup analyses to identify the source of heterogeneity and ensure the stability of the results. Finally, we attempted to minimize publication bias by manually searching for abstracts and letters, and excluding studies with considerable population overlap (>50%), and no significant publication bias was detected by funnel plot or the Egger’s test.

However, there were some limitations that should be noted when interpreting the results. First, the balance of baseline characteristics of CVT cases and controls varied in the included studies. Not all studies matched participants for age and sex, which likely introduced a certain degree of heterogeneity. We attempted to overcome this limitation by performing subgroup analysis and the results were generally similar to the overall results. Second, we were unable to extract data for adults or children separately due to a broad age range of population in most of the studies. Therefore, our analysis failed to adequately control for age and included adults and children of all ancestries. However, we conducted a sensitivity analysis for the overall results, which showed good stability. Third, we did not distinguish between provoked and unprovoked thrombotic factors for CVT as we were unable to obtain these data. Finally, our subgroup analysis according to countries did not take into account different ethnicities within a country, which may make the interpretation of the results difficult.

In conclusion, our meta-analysis confirmed a significant association between FVL and CVT. However, this association was highly dependent on the geography of origin. More studies are warranted to evaluate the prevalence of FVL in unprovoked or provoked CVT. Moreover, the potential role of FVL as a predictor of recurrent CVT also deserves further evaluation.

## Supporting information

S1 File PRISMA 2009 checklist. (DOC)

S2 File Detailed search strategy. (DOC)

S3 File References of 39 studies included in the meta-analysis. (DOC)

S1 Table Meta-analysis on genetic association studies form. (DOC)

S2 Table Characteristics of 8 studies excluded from the meta-analysis. (DOC)
