# Supplementary material for: Prevalence and geographical variation of Factor V Leiden in patients with cerebral venous thrombosis: A meta-analysis
Source: PLoS One. 2018 Aug 29;13(8):e0203309. doi: 10.1371/journal.pone.0203309 (PMC6114929; doi:10.1371/journal.pone.0203309)
Supplement: S2 File — (DOC) [file pone.0203309.s002.doc]

Detailed search strategy.

Search strategy for PubMed

#1 (((factor v) OR factor v leiden) OR G1691A) OR 1691——12814

#2 (((((cerebral venous thrombosis) OR cerebral vein thrombosis) OR cerebral sinus thrombosis) OR intracranial venous thrombosis) OR intracranial vein thrombosis) OR intracranial sinus thrombosis——10426

#1 AND #2 (((((((cerebral venous thrombosis) OR cerebral vein thrombosis) OR cerebral sinus thrombosis) OR intracranial venous thrombosis) OR intracranial vein thrombosis) OR intracranial sinus thrombosis)) AND ((((factor v) OR factor v leiden) OR G1691A) OR 1691)——290

Search strategy for Web of Science

(((((factor v OR factor v leiden) OR g1692a) OR 1691) *AND* (((((cerebral venous thrombosis OR cerebral vein thrombosis) OR cerebral sinus thrombosis) OR intracranial venous thrombosis) OR intracranial vein thrombosis) OR intracranial sinus thrombosis)) *AND* ((((case-control study OR observational study) OR cohort study) OR retrospective study) OR prospective study))——240

Search strategy for Embase

#1 ‘blood clotting factor 5 leiden’/exp OR ‘blood clotting factor 5’/exp OR ‘g1691a’ OR ‘1691’——22515

#2 ‘cerebral vein thrombosis’/exp OR ‘intracranial venous thrombosis’ OR ‘cerebral sinus thrombosis’/exp OR ‘intracranial vein thrombosis’——7735

#3 #1 AND #2——258

#4 #3 AND (‘case control study’/de OR ‘clinical trial’/de OR ‘clinical study’/de OR ‘controlled clinical trial’/de OR ‘controlled study’/de OR ‘multicenter study’/de OR ‘observational study’/de OR ‘prospective study’/de OR ‘randomized controlled trial(topic)’/de OR ‘retrospective study’/de)——86

Search strategy for Cochrane Center Register of Controlled Trials(CENTRAL)

(factor v OR factor v leiden OR G1691A OR 1691) *AND* (cerebral venous thrombosis OR cerebral vein thrombosis OR cerebral sinus thrombosis OR intracranial venous thrombosis OR intracranial vein thrombosis OR intracranial sinus thrombosis)——26
