# Supplementary material for: Prevalence and geographical variation of Factor V Leiden in patients with cerebral venous thrombosis: A meta-analysis
Source: PLoS One. 2018 Aug 29;13(8):e0203309. doi: 10.1371/journal.pone.0203309 (PMC6114929; doi:10.1371/journal.pone.0203309)
Supplement: S3 File — (DOC) [file pone.0203309.s003.doc]

1. [Boncoraglio G](https://www.ncbi.nlm.nih.gov/pubmed/?term=Boncoraglio G%5BAuthor%5D&cauthor=true&cauthor_uid=15171737), [Carriero MR](https://www.ncbi.nlm.nih.gov/pubmed/?term=Carriero MR%5BAuthor%5D&cauthor=true&cauthor_uid=15171737), [Chiapparini L](https://www.ncbi.nlm.nih.gov/pubmed/?term=Chiapparini L%5BAuthor%5D&cauthor=true&cauthor_uid=15171737), [Ciceri E](https://www.ncbi.nlm.nih.gov/pubmed/?term=Ciceri E%5BAuthor%5D&cauthor=true&cauthor_uid=15171737), [Ciusani E](https://www.ncbi.nlm.nih.gov/pubmed/?term=Ciusani E%5BAuthor%5D&cauthor=true&cauthor_uid=15171737), [Erbetta A](https://www.ncbi.nlm.nih.gov/pubmed/?term=Erbetta A%5BAuthor%5D&cauthor=true&cauthor_uid=15171737), et al. Hyperhomocysteinemia and other thrombophilic risk factors in 26 patients with cerebral venousthrombosis.[*Eur J Neurol.*](https://www.ncbi.nlm.nih.gov/pubmed/?term=Hyperhomocysteinemia+and+other+thrombophilic+risk+factors+in+26+patients+with+cerebral+venous+thrombosis) 2004;11:405-409.
2. [Colaizzo D](https://www.ncbi.nlm.nih.gov/pubmed/?term=Colaizzo D%5BAuthor%5D&cauthor=true&cauthor_uid=17307838), [Amitrano L](https://www.ncbi.nlm.nih.gov/pubmed/?term=Amitrano L%5BAuthor%5D&cauthor=true&cauthor_uid=17307838), [Iannaccone L](https://www.ncbi.nlm.nih.gov/pubmed/?term=Iannaccone L%5BAuthor%5D&cauthor=true&cauthor_uid=17307838), [Vergura P](https://www.ncbi.nlm.nih.gov/pubmed/?term=Vergura P%5BAuthor%5D&cauthor=true&cauthor_uid=17307838), [Cappucci F](https://www.ncbi.nlm.nih.gov/pubmed/?term=Cappucci F%5BAuthor%5D&cauthor=true&cauthor_uid=17307838), [Grandone E](https://www.ncbi.nlm.nih.gov/pubmed/?term=Grandone E%5BAuthor%5D&cauthor=true&cauthor_uid=17307838), et al. Gain-of-function gene mutations and venous thromboembolism: distinct roles in different clinicalsettings. [*J Med Genet.*](https://www.ncbi.nlm.nih.gov/pubmed/?term=Gain-of-function+gene+mutations+and+venous+thromboembolism%3A+distinct+roles+in+different+clinical+settings) 2007;44:412-416.
3. [Madonna P](https://www.ncbi.nlm.nih.gov/pubmed/?term=Madonna P%5BAuthor%5D&cauthor=true&cauthor_uid=10884490), [De Stefano V](https://www.ncbi.nlm.nih.gov/pubmed/?term=De Stefano V%5BAuthor%5D&cauthor=true&cauthor_uid=10884490), [Coppola A](https://www.ncbi.nlm.nih.gov/pubmed/?term=Coppola A%5BAuthor%5D&cauthor=true&cauthor_uid=10884490), [Albisinni R](https://www.ncbi.nlm.nih.gov/pubmed/?term=Albisinni R%5BAuthor%5D&cauthor=true&cauthor_uid=10884490), [Cerbone AM](https://www.ncbi.nlm.nih.gov/pubmed/?term=Cerbone AM%5BAuthor%5D&cauthor=true&cauthor_uid=10884490).G20210A PRTH gene mutation and other trombophilic polymorphisms in patients with cerebralvein thrombosis. [*Stroke.*](https://www.ncbi.nlm.nih.gov/pubmed/?term=G20210A+PRTH+Gene+Mutation+and+Other+Trombophilic+Polymorphisms+in+Patients+With+Cerebral+Vein+Thrombosis) 2000;31:1787-1788.
4. [Margaglione M](https://www.ncbi.nlm.nih.gov/pubmed/?term=Margaglione M%5BAuthor%5D&cauthor=true&cauthor_uid=11418373), [Brancaccio V](https://www.ncbi.nlm.nih.gov/pubmed/?term=Brancaccio V%5BAuthor%5D&cauthor=true&cauthor_uid=11418373), [Ciampa A](https://www.ncbi.nlm.nih.gov/pubmed/?term=Ciampa A%5BAuthor%5D&cauthor=true&cauthor_uid=11418373), [Papa ML](https://www.ncbi.nlm.nih.gov/pubmed/?term=Papa ML%5BAuthor%5D&cauthor=true&cauthor_uid=11418373), [Grandone E](https://www.ncbi.nlm.nih.gov/pubmed/?term=Grandone E%5BAuthor%5D&cauthor=true&cauthor_uid=11418373), [Di Minno G](https://www.ncbi.nlm.nih.gov/pubmed/?term=Di Minno G%5BAuthor%5D&cauthor=true&cauthor_uid=11418373). Inherited thrombophilic risk factors in large cohort of individuals referred to Italianthrombophilia centers: distinct roles in different clinical settings. [*Haematologica.*](https://www.ncbi.nlm.nih.gov/pubmed/?term=Inherited+thrombophilic+risk+factors+in+a+large+cohort+of+individuals+referred+to+Italian+thrombophilia+centers%3A+distinct+roles+in+different+clinical+settings) 2001;86:634-639.
5. [Martinelli I](https://www.ncbi.nlm.nih.gov/pubmed/?term=Martinelli I%5BAuthor%5D&cauthor=true&cauthor_uid=12714502), [Battaglioli T](https://www.ncbi.nlm.nih.gov/pubmed/?term=Battaglioli T%5BAuthor%5D&cauthor=true&cauthor_uid=12714502), [Pedotti P](https://www.ncbi.nlm.nih.gov/pubmed/?term=Pedotti P%5BAuthor%5D&cauthor=true&cauthor_uid=12714502), [Cattaneo M](https://www.ncbi.nlm.nih.gov/pubmed/?term=Cattaneo M%5BAuthor%5D&cauthor=true&cauthor_uid=12714502), [Mannucci PM](https://www.ncbi.nlm.nih.gov/pubmed/?term=Mannucci PM%5BAuthor%5D&cauthor=true&cauthor_uid=12714502). Hyperhomocysteinemia in cerebral vein thrombosis. [*Blood.*](https://www.ncbi.nlm.nih.gov/pubmed/12714502) 2003;102:1363-1366.
6. [Tufano A](https://www.ncbi.nlm.nih.gov/pubmed/?term=Tufano A%5BAuthor%5D&cauthor=true&cauthor_uid=23399370), [Guida A](https://www.ncbi.nlm.nih.gov/pubmed/?term=Guida A%5BAuthor%5D&cauthor=true&cauthor_uid=23399370), [Coppola A](https://www.ncbi.nlm.nih.gov/pubmed/?term=Coppola A%5BAuthor%5D&cauthor=true&cauthor_uid=23399370), [Nardo A](https://www.ncbi.nlm.nih.gov/pubmed/?term=Nardo A%5BAuthor%5D&cauthor=true&cauthor_uid=23399370), [Di Capua M](https://www.ncbi.nlm.nih.gov/pubmed/?term=Di Capua M%5BAuthor%5D&cauthor=true&cauthor_uid=23399370), [Quintavalle G](https://www.ncbi.nlm.nih.gov/pubmed/?term=Quintavalle G%5BAuthor%5D&cauthor=true&cauthor_uid=23399370), et al. Risk factors and recurrent thrombotic episodes in patients with cerebral venous thrombosis. [*Blood Transfus.*](https://www.ncbi.nlm.nih.gov/pubmed/23399370) 2014;12 Suppl 1:s337-42.
7. [Ventura P](https://www.ncbi.nlm.nih.gov/pubmed/?term=Ventura P%5BAuthor%5D&cauthor=true&cauthor_uid=14707415), [Cobelli M](https://www.ncbi.nlm.nih.gov/pubmed/?term=Cobelli M%5BAuthor%5D&cauthor=true&cauthor_uid=14707415), [Marietta M](https://www.ncbi.nlm.nih.gov/pubmed/?term=Marietta M%5BAuthor%5D&cauthor=true&cauthor_uid=14707415), [Panini R](https://www.ncbi.nlm.nih.gov/pubmed/?term=Panini R%5BAuthor%5D&cauthor=true&cauthor_uid=14707415), [Rosa MC](https://www.ncbi.nlm.nih.gov/pubmed/?term=Rosa MC%5BAuthor%5D&cauthor=true&cauthor_uid=14707415), [Salvioli G](https://www.ncbi.nlm.nih.gov/pubmed/?term=Salvioli G%5BAuthor%5D&cauthor=true&cauthor_uid=14707415). Hyperhomocysteinemia and other newly recognized inherited coagulation disorders (factor VLeiden and prothrombin gene mutation)in patients with idiopathic cerebral vein thrombosis. [*Cerebrovasc Dis.*](https://www.ncbi.nlm.nih.gov/pubmed/?term=Hyperhomocysteinemia+and+Other+Newly+Recognized+Inherited+Coagulation+Disorders+(Factor+V+Leiden+and+Prothrombin+Gene+Mutation)+in+Patients+with+Idiopathic+Cerebral+Vein+Thrombosis) 2004;17:153-159.
8. [Beye A](https://www.ncbi.nlm.nih.gov/pubmed/?term=Beye A%5BAuthor%5D&cauthor=true&cauthor_uid=28869458), [Pindur G](https://www.ncbi.nlm.nih.gov/pubmed/?term=Pindur G%5BAuthor%5D&cauthor=true&cauthor_uid=28869458). Clinical significance of factor V leiden and prothrombin G20210A-mutations in cerebral venous thrombosis - comparison with arterial ischemic stroke. [*Clin Hemorheol Microcirc.*](https://www.ncbi.nlm.nih.gov/pubmed/?term=Clinical+significance+of+factor+V+leiden+and+1+prothrombin+G20210A-mutations+in+2+cerebral+venous+thrombosis) 2017; 67:261-266.
9. Heller C, [Heinecke A](https://www.ncbi.nlm.nih.gov/pubmed/?term=Heinecke A%5BAuthor%5D&cauthor=true&cauthor_uid=12939214), [Junker R](https://www.ncbi.nlm.nih.gov/pubmed/?term=Junker R%5BAuthor%5D&cauthor=true&cauthor_uid=12939214), [Knöfler R](https://www.ncbi.nlm.nih.gov/pubmed/?term=Knöfler R%5BAuthor%5D&cauthor=true&cauthor_uid=12939214), [Kosch A](https://www.ncbi.nlm.nih.gov/pubmed/?term=Kosch A%5BAuthor%5D&cauthor=true&cauthor_uid=12939214), [Kurnik K](https://www.ncbi.nlm.nih.gov/pubmed/?term=Kurnik K%5BAuthor%5D&cauthor=true&cauthor_uid=12939214), et al. Cerebral venous thrombosis in children: a multifactorial origin. [*Circulation.*](https://www.ncbi.nlm.nih.gov/pubmed/12939214) 2003;108:1362-1367.
10. [Junker R](https://www.ncbi.nlm.nih.gov/pubmed/?term=Junker R%5BAuthor%5D&cauthor=true&cauthor_uid=9798998), [Nabavi DG](https://www.ncbi.nlm.nih.gov/pubmed/?term=Nabavi DG%5BAuthor%5D&cauthor=true&cauthor_uid=9798998), [Wolff E](https://www.ncbi.nlm.nih.gov/pubmed/?term=Wolff E%5BAuthor%5D&cauthor=true&cauthor_uid=9798998), [Lüdemann P](https://www.ncbi.nlm.nih.gov/pubmed/?term=Lüdemann P%5BAuthor%5D&cauthor=true&cauthor_uid=9798998), [Nowak-Göttl U](https://www.ncbi.nlm.nih.gov/pubmed/?term=Nowak-Göttl U%5BAuthor%5D&cauthor=true&cauthor_uid=9798998), [Käse M](https://www.ncbi.nlm.nih.gov/pubmed/?term=Käse M%5BAuthor%5D&cauthor=true&cauthor_uid=9798998), et al. Plasminogen activator inhibitor-1 4G/4G-genotype is associated with cerebral sinus thrombosis in factor V Leiden carriers. [*Thromb Haemost.*](https://www.ncbi.nlm.nih.gov/pubmed/9798998) 1998;80:706-707.
11. [Lichy C](https://www.ncbi.nlm.nih.gov/pubmed/?term=Lichy C%5BAuthor%5D&cauthor=true&cauthor_uid=16155788), [Dong-Si T](https://www.ncbi.nlm.nih.gov/pubmed/?term=Dong-Si T%5BAuthor%5D&cauthor=true&cauthor_uid=16155788), [Reuner K](https://www.ncbi.nlm.nih.gov/pubmed/?term=Reuner K%5BAuthor%5D&cauthor=true&cauthor_uid=16155788), [Genius J](https://www.ncbi.nlm.nih.gov/pubmed/?term=Genius J%5BAuthor%5D&cauthor=true&cauthor_uid=16155788), [Rickmann H](https://www.ncbi.nlm.nih.gov/pubmed/?term=Rickmann H%5BAuthor%5D&cauthor=true&cauthor_uid=16155788), [Hampe T](https://www.ncbi.nlm.nih.gov/pubmed/?term=Hampe T%5BAuthor%5D&cauthor=true&cauthor_uid=16155788), et al. Risk of cerebral venous thrombosis and novel gene polymorphisms ofthe coagulation and fibrinolytic systems. [*J Neurol.*](https://www.ncbi.nlm.nih.gov/pubmed/?term=Risk+of+cerebral+venous+thrombosis+and+novel+gene+polymorphisms+of+the+coagulation+and+fibrinolytic+systems) 2005;253:316-320.
12. [Lüdemann P](https://www.ncbi.nlm.nih.gov/pubmed/?term=Lüdemann P%5BAuthor%5D&cauthor=true&cauthor_uid=9836759), [Nabavi DG](https://www.ncbi.nlm.nih.gov/pubmed/?term=Nabavi DG%5BAuthor%5D&cauthor=true&cauthor_uid=9836759), [Junker R](https://www.ncbi.nlm.nih.gov/pubmed/?term=Junker R%5BAuthor%5D&cauthor=true&cauthor_uid=9836759), [Wolff E](https://www.ncbi.nlm.nih.gov/pubmed/?term=Wolff E%5BAuthor%5D&cauthor=true&cauthor_uid=9836759), [Papke K](https://www.ncbi.nlm.nih.gov/pubmed/?term=Papke K%5BAuthor%5D&cauthor=true&cauthor_uid=9836759), [Buchner H](https://www.ncbi.nlm.nih.gov/pubmed/?term=Buchner H%5BAuthor%5D&cauthor=true&cauthor_uid=9836759), et al. Factor V Leiden mutation is a risk factor for cerebral venous thrombosis: a case-control study of 55 patients. [*Stroke.*](https://www.ncbi.nlm.nih.gov/pubmed/?term=Factor+V+Leiden+Mutation+Is+a+Risk+Factor+for+Cerebral+Venous+Thrombosis+A+Case-Control+Study+of+55+Patients) 1998;29:2507-2510.
13. [Ringelstein M](https://www.ncbi.nlm.nih.gov/pubmed/?term=Ringelstein M%5BAuthor%5D&cauthor=true&cauthor_uid=22527222), [Jung A](https://www.ncbi.nlm.nih.gov/pubmed/?term=Jung A%5BAuthor%5D&cauthor=true&cauthor_uid=22527222), [Berger K](https://www.ncbi.nlm.nih.gov/pubmed/?term=Berger K%5BAuthor%5D&cauthor=true&cauthor_uid=22527222), [Stoll M](https://www.ncbi.nlm.nih.gov/pubmed/?term=Stoll M%5BAuthor%5D&cauthor=true&cauthor_uid=22527222), [Madlener K](https://www.ncbi.nlm.nih.gov/pubmed/?term=Madlener K%5BAuthor%5D&cauthor=true&cauthor_uid=22527222), [Klötzsch C](https://www.ncbi.nlm.nih.gov/pubmed/?term=Klötzsch C%5BAuthor%5D&cauthor=true&cauthor_uid=22527222), et al. Promotor polymorphisms of plasminogen activator inhibitor-1 and other thrombophilic genotypes in cerebral venous thrombosis:a case-control study in adults. [*J Neurol.*](https://www.ncbi.nlm.nih.gov/pubmed/?term=Promotor+polymorphisms+of+plasminogen+activator+inhibitor-1+and+other+thrombophilic+genotypes+in+cerebral+venous+thrombosis%3A+a+case-control+study+in+adults) 2012;259:2287-2292.
14. [Schobess R](https://www.ncbi.nlm.nih.gov/pubmed/?term=Schobess R%5BAuthor%5D&cauthor=true&cauthor_uid=10650846), [Junker R](https://www.ncbi.nlm.nih.gov/pubmed/?term=Junker R%5BAuthor%5D&cauthor=true&cauthor_uid=10650846), [Auberger K](https://www.ncbi.nlm.nih.gov/pubmed/?term=Auberger K%5BAuthor%5D&cauthor=true&cauthor_uid=10650846), [Münchow N](https://www.ncbi.nlm.nih.gov/pubmed/?term=Münchow N%5BAuthor%5D&cauthor=true&cauthor_uid=10650846), [Burdach S](https://www.ncbi.nlm.nih.gov/pubmed/?term=Burdach S%5BAuthor%5D&cauthor=true&cauthor_uid=10650846), [Nowak-Göttl U](https://www.ncbi.nlm.nih.gov/pubmed/?term=Nowak-Göttl U%5BAuthor%5D&cauthor=true&cauthor_uid=10650846). Factor V G1691A and prothrombin G20210A in childhood spontaneous venous thrombosis--evidence of an age-dependent thrombotic onset in carriers of factor V G1691A and prothrombinG20210A mutation. [*Eur J Pediatr.*](https://www.ncbi.nlm.nih.gov/pubmed/?term=Factor+V+G1691A+and+prothrombin+G20210A+in+childhood+spontaneous+venous+thrombosis+–+Evidence+of+an+age-dependent+thrombotic+onset+in+carriers+of+factor+V+G1691A+and+prothrombin+G20210A+mutation) 1999;158 Suppl 3:S105-S108.
15. [Stolz E](https://www.ncbi.nlm.nih.gov/pubmed/?term=Stolz E%5BAuthor%5D&cauthor=true&cauthor_uid=17450317), [Valdueza JM](https://www.ncbi.nlm.nih.gov/pubmed/?term=Valdueza JM%5BAuthor%5D&cauthor=true&cauthor_uid=17450317), [Grebe M](https://www.ncbi.nlm.nih.gov/pubmed/?term=Grebe M%5BAuthor%5D&cauthor=true&cauthor_uid=17450317), [Schlachetzki F](https://www.ncbi.nlm.nih.gov/pubmed/?term=Schlachetzki F%5BAuthor%5D&cauthor=true&cauthor_uid=17450317), [Schmitt E](https://www.ncbi.nlm.nih.gov/pubmed/?term=Schmitt E%5BAuthor%5D&cauthor=true&cauthor_uid=17450317), [Madlener K](https://www.ncbi.nlm.nih.gov/pubmed/?term=Madlener K%5BAuthor%5D&cauthor=true&cauthor_uid=17450317), et al. Anemia as a risk factor for cerebral venous thrombosis? An old hypothesis revisited. Results of a prospective study. [*J Neurol.*](https://www.ncbi.nlm.nih.gov/pubmed/?term=Anemia+as+a+risk+factor+for+cerebral+venous+thrombosis%3F+An+old+hypothesis+revisited+Results+of+a+prospective+study) 2007;254:729-734.
16. [Weih M](https://www.ncbi.nlm.nih.gov/pubmed/?term=Weih M%5BAuthor%5D&cauthor=true&cauthor_uid=9553844), [Vetter B](https://www.ncbi.nlm.nih.gov/pubmed/?term=Vetter B%5BAuthor%5D&cauthor=true&cauthor_uid=9553844), [Ziemer S](https://www.ncbi.nlm.nih.gov/pubmed/?term=Ziemer S%5BAuthor%5D&cauthor=true&cauthor_uid=9553844), [Mehraein S](https://www.ncbi.nlm.nih.gov/pubmed/?term=Mehraein S%5BAuthor%5D&cauthor=true&cauthor_uid=9553844), [Valdueza JM](https://www.ncbi.nlm.nih.gov/pubmed/?term=Valdueza JM%5BAuthor%5D&cauthor=true&cauthor_uid=9553844), [Koscielny J](https://www.ncbi.nlm.nih.gov/pubmed/?term=Koscielny J%5BAuthor%5D&cauthor=true&cauthor_uid=9553844), et al. Increased rate of factor V Leiden mutation in patients with cerebral venous thrombosis. [*J Neurol.*](https://www.ncbi.nlm.nih.gov/pubmed/?term=Increased+rate+of+factor+V+Leiden+S.+Z+emer+mutation+in+patients+with+cerebral+J.+Mehradueza+ein+venous+thrombosis.+1998+Weih) 1998;245:149-152.
17. [Le Cam-Duchez V](https://www.ncbi.nlm.nih.gov/pubmed/?term=Le Cam-Duchez V%5BAuthor%5D&cauthor=true&cauthor_uid=18677630), [Bagan-Triquenot A](https://www.ncbi.nlm.nih.gov/pubmed/?term=Bagan-Triquenot A%5BAuthor%5D&cauthor=true&cauthor_uid=18677630), [Barbay V](https://www.ncbi.nlm.nih.gov/pubmed/?term=Barbay V%5BAuthor%5D&cauthor=true&cauthor_uid=18677630), [Mihout B](https://www.ncbi.nlm.nih.gov/pubmed/?term=Mihout B%5BAuthor%5D&cauthor=true&cauthor_uid=18677630), [Borg JY](https://www.ncbi.nlm.nih.gov/pubmed/?term=Borg JY%5BAuthor%5D&cauthor=true&cauthor_uid=18677630). The G79A polymorphism of protein Z gene is an independent risk factor for cerebral venousthrombosis. [*J Neurol.*](https://www.ncbi.nlm.nih.gov/pubmed/?term=The+G79A+polymorphism+of+protein+Z+gene+is+an+independent+risk+factor+for+cerebral+venous+thrombosis) 2008;255:1521-1525.
18. [Zuber M](https://www.ncbi.nlm.nih.gov/pubmed/?term=Zuber M%5BAuthor%5D&cauthor=true&cauthor_uid=8841317), [Toulon P](https://www.ncbi.nlm.nih.gov/pubmed/?term=Toulon P%5BAuthor%5D&cauthor=true&cauthor_uid=8841317), [Marnet L](https://www.ncbi.nlm.nih.gov/pubmed/?term=Marnet L%5BAuthor%5D&cauthor=true&cauthor_uid=8841317), [Mas JL](https://www.ncbi.nlm.nih.gov/pubmed/?term=Mas JL%5BAuthor%5D&cauthor=true&cauthor_uid=8841317). Factor V Leiden mutation in cerebral venous thrombosis. [*Stroke.*](https://www.ncbi.nlm.nih.gov/pubmed/?term=Factor+V+Leiden+Mutation+in+Cerebral+Venous+Thrombosis+Zuber+1996)1996;27:1721-1723.
19. [Gadelha T](https://www.ncbi.nlm.nih.gov/pubmed/?term=Gadelha T%5BAuthor%5D&cauthor=true&cauthor_uid=15528884), [André C](https://www.ncbi.nlm.nih.gov/pubmed/?term=André C%5BAuthor%5D&cauthor=true&cauthor_uid=15528884), [Jucá AA](https://www.ncbi.nlm.nih.gov/pubmed/?term=Jucá AA%5BAuthor%5D&cauthor=true&cauthor_uid=15528884), [Nucci M](https://www.ncbi.nlm.nih.gov/pubmed/?term=Nucci M%5BAuthor%5D&cauthor=true&cauthor_uid=15528884). Prothrombin 20210A and oral contraceptive use as risk factors for cerebral venous thrombosis. [*Cerebrovasc Dis.*](https://www.ncbi.nlm.nih.gov/pubmed/15528884) 2005;19:49-52.
20. [Orikaza CM](https://www.ncbi.nlm.nih.gov/pubmed/?term=Orikaza CM%5BAuthor%5D&cauthor=true&cauthor_uid=24252537), [Morelli VM](https://www.ncbi.nlm.nih.gov/pubmed/?term=Morelli VM%5BAuthor%5D&cauthor=true&cauthor_uid=24252537), [Matos MF](https://www.ncbi.nlm.nih.gov/pubmed/?term=Matos MF%5BAuthor%5D&cauthor=true&cauthor_uid=24252537), [Lourenço DM](https://www.ncbi.nlm.nih.gov/pubmed/?term=Lourenço DM%5BAuthor%5D&cauthor=true&cauthor_uid=24252537). Haplotypes of TAFI gene andthe risk of cerebral venous thrombosis--a case-control study. [*Thromb Res.*](https://www.ncbi.nlm.nih.gov/pubmed/?term=Haplotypes+of+TAFI+gene+and+the+risk+of+cerebral+venous+thrombosis+-+a+case-control+study) 2014;133:120-124.
21. [Rodrigues CA](https://www.ncbi.nlm.nih.gov/pubmed/?term=Rodrigues CA%5BAuthor%5D&cauthor=true&cauthor_uid=15219221), [Rocha LK](https://www.ncbi.nlm.nih.gov/pubmed/?term=Rocha LK%5BAuthor%5D&cauthor=true&cauthor_uid=15219221), [Morelli VM](https://www.ncbi.nlm.nih.gov/pubmed/?term=Morelli VM%5BAuthor%5D&cauthor=true&cauthor_uid=15219221), [Franco RF](https://www.ncbi.nlm.nih.gov/pubmed/?term=Franco RF%5BAuthor%5D&cauthor=true&cauthor_uid=15219221), [Lourenço DM](https://www.ncbi.nlm.nih.gov/pubmed/?term=Lourenço DM%5BAuthor%5D&cauthor=true&cauthor_uid=15219221). Prothrombin G20210A mutation, and not factor V Leiden mutation, is a risk factor for cerebral venous thrombosis in Brazilian patients. [*J Thromb Haemost.*](https://www.ncbi.nlm.nih.gov/pubmed/15219221)2004;2:1211-1212.
22. [Voetsch B](https://www.ncbi.nlm.nih.gov/pubmed/?term=Voetsch B%5BAuthor%5D&cauthor=true&cauthor_uid=10739378), [Damasceno BP](https://www.ncbi.nlm.nih.gov/pubmed/?term=Damasceno BP%5BAuthor%5D&cauthor=true&cauthor_uid=10739378), [Camargo EC](https://www.ncbi.nlm.nih.gov/pubmed/?term=Camargo EC%5BAuthor%5D&cauthor=true&cauthor_uid=10739378), [Massaro A](https://www.ncbi.nlm.nih.gov/pubmed/?term=Massaro A%5BAuthor%5D&cauthor=true&cauthor_uid=10739378), [Bacheschi LA](https://www.ncbi.nlm.nih.gov/pubmed/?term=Bacheschi LA%5BAuthor%5D&cauthor=true&cauthor_uid=10739378), [Scaff M](https://www.ncbi.nlm.nih.gov/pubmed/?term=Scaff M%5BAuthor%5D&cauthor=true&cauthor_uid=10739378), et al. Inherited thrombophilia as a risk factor for the development of ischemic stroke in young adults. [*Thromb Haemost.*](https://www.ncbi.nlm.nih.gov/pubmed/?term=Inherited+Thrombophilia+as+a+Risk+Factor+for+the+Development+of+Ischemic+Stroke+in+Young+Adults)2000;83:229-233.
23. [N Ashjazadeh](http://xueshu.baidu.com/s?wd=author%3A(Nahid Ashjazadeh) &tn=SE_baiduxueshu_c1gjeupa&ie=utf-8&sc_f_para=sc_hilight%3Dperson)， [M Poursadeghfard](http://xueshu.baidu.com/s?wd=author%3A(Maryam Poursadeghfard) &tn=SE_baiduxueshu_c1gjeupa&ie=utf-8&sc_f_para=sc_hilight%3Dperson)， [S Farjadian](http://xueshu.baidu.com/s?wd=author%3A(Shirin Farjadian) &tn=SE_baiduxueshu_c1gjeupa&ie=utf-8&sc_f_para=sc_hilight%3Dperson). Factor V G1691A and prothrombin G20210A gene polymorphisms among Iranian patients with cerebral venous thrombosis. [Neurology Asia.](http://xueshu.baidu.com/usercenter/data/journal?cmd=jump&wd=journaluri%3A(3cf81343f8d8fd3e) 《Neurology Asia》&tn=SE_baiduxueshu_c1gjeupa&ie=utf-8&sc_f_para=sc_hilight%3Dpublish&sort=sc_cited)2012;17:199-203.
24. [Rahimi Z](https://www.ncbi.nlm.nih.gov/pubmed/?term=Rahimi Z%5BAuthor%5D&cauthor=true&cauthor_uid=19703820), [Mozafari H](https://www.ncbi.nlm.nih.gov/pubmed/?term=Mozafari H%5BAuthor%5D&cauthor=true&cauthor_uid=19703820), [Bigvand AH](https://www.ncbi.nlm.nih.gov/pubmed/?term=Bigvand AH%5BAuthor%5D&cauthor=true&cauthor_uid=19703820), [Doulabi RM](https://www.ncbi.nlm.nih.gov/pubmed/?term=Doulabi RM%5BAuthor%5D&cauthor=true&cauthor_uid=19703820), [Vaisi-Raygani A](https://www.ncbi.nlm.nih.gov/pubmed/?term=Vaisi-Raygani A%5BAuthor%5D&cauthor=true&cauthor_uid=19703820), [Afshari D](https://www.ncbi.nlm.nih.gov/pubmed/?term=Afshari D%5BAuthor%5D&cauthor=true&cauthor_uid=19703820), et al. Cerebral venous and sinus thrombosis and thrombophilic mutations in Western Iran: association with factor V Leiden. [*Clin Appl Thromb Hemost.*](https://www.ncbi.nlm.nih.gov/pubmed/?term=Cerebral+Venous+and+Sinus+Thrombosis+and+Thrombophilic+Mutations+in+Western+Iran%3A+Association+With+Factor+V+Leiden) 2010;16:430-434.
25. Saadatnia M, [Salehi M](https://www.ncbi.nlm.nih.gov/pubmed/?term=Salehi M%5BAuthor%5D&cauthor=true&cauthor_uid=26600830), [Movahedian A](https://www.ncbi.nlm.nih.gov/pubmed/?term=Movahedian A%5BAuthor%5D&cauthor=true&cauthor_uid=26600830), [Shariat SZ](https://www.ncbi.nlm.nih.gov/pubmed/?term=Shariat SZ%5BAuthor%5D&cauthor=true&cauthor_uid=26600830), [Salari M](https://www.ncbi.nlm.nih.gov/pubmed/?term=Salari M%5BAuthor%5D&cauthor=true&cauthor_uid=26600830), [Tajmirriahi M](https://www.ncbi.nlm.nih.gov/pubmed/?term=Tajmirriahi M%5BAuthor%5D&cauthor=true&cauthor_uid=26600830), et al. Factor V Leiden, factor V Cambridge, factor II GA20210, and methylenetetrahydrofolate reductase in cerebral venous and sinus thrombosis: A case-control study. [*J Res Med Sci.*](https://www.ncbi.nlm.nih.gov/pubmed/?term=Factor+V+Leiden%2C+factor+V+Cambridge%2C+factor+II+GA20210%2C+and+methylenetetrahydrofolate+reductase+in+cerebral+venous+and+sinus+thrombosis%3A+A+case-control+study) 2015;20:554-562.
26. [Ben Salem-Berrabah O](https://www.ncbi.nlm.nih.gov/pubmed/?term=Ben Salem-Berrabah O%5BAuthor%5D&cauthor=true&cauthor_uid=22721898), [Fekih-Mrissa N](https://www.ncbi.nlm.nih.gov/pubmed/?term=Fekih-Mrissa N%5BAuthor%5D&cauthor=true&cauthor_uid=22721898), [N'siri B](https://www.ncbi.nlm.nih.gov/pubmed/?term=N'siri B%5BAuthor%5D&cauthor=true&cauthor_uid=22721898), [Ben Hamida A](https://www.ncbi.nlm.nih.gov/pubmed/?term=Ben Hamida A%5BAuthor%5D&cauthor=true&cauthor_uid=22721898), [Benammar-Elgaaied A](https://www.ncbi.nlm.nih.gov/pubmed/?term=Benammar-Elgaaied A%5BAuthor%5D&cauthor=true&cauthor_uid=22721898), [Gritli N](https://www.ncbi.nlm.nih.gov/pubmed/?term=Gritli N%5BAuthor%5D&cauthor=true&cauthor_uid=22721898), et al. Thrombophilic polymorphisms - factor V Leiden G1691A, prothrombin G20210A and MTHFRC677T - in Tunisian patients with cerebral venous thrombosis. [*J Clin Neurosci.*](https://www.ncbi.nlm.nih.gov/pubmed/?term=Thrombophilic+polymorphisms+–+factor+V+Leiden+G1691A%2C+prothrombin+G20210A+and+MTHFR+C677T+–+in+Tunisian+patients+with+cerebral+venous+thrombosis) 2012;19:1326-1327.
27. [Klai S](https://www.ncbi.nlm.nih.gov/pubmed/?term=Klai S%5BAuthor%5D&cauthor=true&cauthor_uid=23337711), [Fekih-Mrissa N](https://www.ncbi.nlm.nih.gov/pubmed/?term=Fekih-Mrissa N%5BAuthor%5D&cauthor=true&cauthor_uid=23337711), [Mrissa R](https://www.ncbi.nlm.nih.gov/pubmed/?term=Mrissa R%5BAuthor%5D&cauthor=true&cauthor_uid=23337711), [Rachdi R](https://www.ncbi.nlm.nih.gov/pubmed/?term=Rachdi R%5BAuthor%5D&cauthor=true&cauthor_uid=23337711), [Gritli N](https://www.ncbi.nlm.nih.gov/pubmed/?term=Gritli N%5BAuthor%5D&cauthor=true&cauthor_uid=23337711). Maternal cerebral venous thrombosis, uncommon but serious disorder, pathologic predictors and contribution of prothrombotic abnormalities. [*Blood Coagul Fibrinolysis.*](https://www.ncbi.nlm.nih.gov/pubmed/?term=Maternal+cerebral+venous+thrombosis%2C+uncommon+but+serious+disorder%2C+pathologic+predictors+and+contribution+of+prothrombotic+abnormalities) 2013;24:269-272.
28. [Hagstrom JN](https://www.ncbi.nlm.nih.gov/pubmed/?term=Hagstrom JN%5BAuthor%5D&cauthor=true&cauthor_uid=9842043), [Walter J](https://www.ncbi.nlm.nih.gov/pubmed/?term=Walter J%5BAuthor%5D&cauthor=true&cauthor_uid=9842043), [Bluebond-Langner R](https://www.ncbi.nlm.nih.gov/pubmed/?term=Bluebond-Langner R%5BAuthor%5D&cauthor=true&cauthor_uid=9842043), [Amatniek JC](https://www.ncbi.nlm.nih.gov/pubmed/?term=Amatniek JC%5BAuthor%5D&cauthor=true&cauthor_uid=9842043), [Manno CS](https://www.ncbi.nlm.nih.gov/pubmed/?term=Manno CS%5BAuthor%5D&cauthor=true&cauthor_uid=9842043), [High KA](https://www.ncbi.nlm.nih.gov/pubmed/?term=High KA%5BAuthor%5D&cauthor=true&cauthor_uid=9842043). Prevalence of the factor V leiden mutation in children and neonates with thromboembolic disease. [*J Pediatr.*](https://www.ncbi.nlm.nih.gov/pubmed/9842043) 1998;133:777-781.
29. [Miller SP](https://www.ncbi.nlm.nih.gov/pubmed/?term=Miller SP%5BAuthor%5D&cauthor=true&cauthor_uid=17008620), [Wu YW](https://www.ncbi.nlm.nih.gov/pubmed/?term=Wu YW%5BAuthor%5D&cauthor=true&cauthor_uid=17008620), [Lee J](https://www.ncbi.nlm.nih.gov/pubmed/?term=Lee J%5BAuthor%5D&cauthor=true&cauthor_uid=17008620), [Lammer EJ](https://www.ncbi.nlm.nih.gov/pubmed/?term=Lammer EJ%5BAuthor%5D&cauthor=true&cauthor_uid=17008620), [Iovannisci DM](https://www.ncbi.nlm.nih.gov/pubmed/?term=Iovannisci DM%5BAuthor%5D&cauthor=true&cauthor_uid=17008620), [Glidden DV](https://www.ncbi.nlm.nih.gov/pubmed/?term=Glidden DV%5BAuthor%5D&cauthor=true&cauthor_uid=17008620),et al. Candidate gene polymorphisms do not differ between newborns with stroke and normal controls. [*Stroke.*](https://www.ncbi.nlm.nih.gov/pubmed/17008620) 2006;37:2678-2683.
30. [Hillier CE](https://www.ncbi.nlm.nih.gov/pubmed/?term=Hillier CE%5BAuthor%5D&cauthor=true&cauthor_uid=10024925), [Collins PW](https://www.ncbi.nlm.nih.gov/pubmed/?term=Collins PW%5BAuthor%5D&cauthor=true&cauthor_uid=10024925), [Bowen DJ](https://www.ncbi.nlm.nih.gov/pubmed/?term=Bowen DJ%5BAuthor%5D&cauthor=true&cauthor_uid=10024925), [Bowley S](https://www.ncbi.nlm.nih.gov/pubmed/?term=Bowley S%5BAuthor%5D&cauthor=true&cauthor_uid=10024925), [Wiles CM](https://www.ncbi.nlm.nih.gov/pubmed/?term=Wiles CM%5BAuthor%5D&cauthor=true&cauthor_uid=10024925). Inherited prothrombotic risk factors and cerebral venous thrombosis. [*QJM.*](https://www.ncbi.nlm.nih.gov/pubmed/10024925) 1998;91:677-680.
31. [Bombeli T](https://www.ncbi.nlm.nih.gov/pubmed/?term=Bombeli T%5BAuthor%5D&cauthor=true&cauthor_uid=12111785), [Basic A](https://www.ncbi.nlm.nih.gov/pubmed/?term=Basic A%5BAuthor%5D&cauthor=true&cauthor_uid=12111785), [Fehr J](https://www.ncbi.nlm.nih.gov/pubmed/?term=Fehr J%5BAuthor%5D&cauthor=true&cauthor_uid=12111785). Prevalence of hereditary thrombophilia in patients with thrombosis in different venous systems. [*Am J Hematol.*](https://www.ncbi.nlm.nih.gov/pubmed/?term=Bombeli+2002+thrombophilia) 2002;70:126-132.
32. [Bonduel M](https://www.ncbi.nlm.nih.gov/pubmed/?term=Bonduel M%5BAuthor%5D&cauthor=true&cauthor_uid=12749008), [Sciuccati G](https://www.ncbi.nlm.nih.gov/pubmed/?term=Sciuccati G%5BAuthor%5D&cauthor=true&cauthor_uid=12749008), [Hepner M](https://www.ncbi.nlm.nih.gov/pubmed/?term=Hepner M%5BAuthor%5D&cauthor=true&cauthor_uid=12749008), [Pieroni G](https://www.ncbi.nlm.nih.gov/pubmed/?term=Pieroni G%5BAuthor%5D&cauthor=true&cauthor_uid=12749008), [Torres AF](https://www.ncbi.nlm.nih.gov/pubmed/?term=Torres AF%5BAuthor%5D&cauthor=true&cauthor_uid=12749008), [Mardaraz C](https://www.ncbi.nlm.nih.gov/pubmed/?term=Mardaraz C%5BAuthor%5D&cauthor=true&cauthor_uid=12749008), et al. Factor V Leiden and prothrombin gene G20210A mutation in children with cerebralthromboembolism. [*Am J Hematol.*](https://www.ncbi.nlm.nih.gov/pubmed/12749008)2003;73:81-86.
33. [Kenet G](https://www.ncbi.nlm.nih.gov/pubmed/?term=Kenet G%5BAuthor%5D&cauthor=true&cauthor_uid=15467900), [Waldman D](https://www.ncbi.nlm.nih.gov/pubmed/?term=Waldman D%5BAuthor%5D&cauthor=true&cauthor_uid=15467900), [Lubetsky A](https://www.ncbi.nlm.nih.gov/pubmed/?term=Lubetsky A%5BAuthor%5D&cauthor=true&cauthor_uid=15467900), [Kornbrut N](https://www.ncbi.nlm.nih.gov/pubmed/?term=Kornbrut N%5BAuthor%5D&cauthor=true&cauthor_uid=15467900), [Khalil A](https://www.ncbi.nlm.nih.gov/pubmed/?term=Khalil A%5BAuthor%5D&cauthor=true&cauthor_uid=15467900), [Koren A](https://www.ncbi.nlm.nih.gov/pubmed/?term=Koren A%5BAuthor%5D&cauthor=true&cauthor_uid=15467900), et al. Paediatric cerebral sinus vein thrombosis. A multi-center, case-controlled study. [*Thromb Haemost.*](https://www.ncbi.nlm.nih.gov/pubmed/?term=Paediatric+cerebral+sinus+vein+thrombosis+A+multi-center%2C+case-controlled+study) 2004;92:713-718.
34. [Dindagur N](https://www.ncbi.nlm.nih.gov/pubmed/?term=Dindagur N%5BAuthor%5D&cauthor=true&cauthor_uid=16839569), [Kruthika-Vinod TP](https://www.ncbi.nlm.nih.gov/pubmed/?term=Kruthika-Vinod TP%5BAuthor%5D&cauthor=true&cauthor_uid=16839569), [Christopher R](https://www.ncbi.nlm.nih.gov/pubmed/?term=Christopher R%5BAuthor%5D&cauthor=true&cauthor_uid=16839569). Thrombophilic gene polymorphisms in puerperal cerebral veno-sinus thrombosis. [*J Neurol Sci.*](https://www.ncbi.nlm.nih.gov/pubmed/16839569)2006;249:25-30.
35. [Romero A](https://www.ncbi.nlm.nih.gov/pubmed/?term=Romero A%5BAuthor%5D&cauthor=true&cauthor_uid=17537363), [Marco P](https://www.ncbi.nlm.nih.gov/pubmed/?term=Marco P%5BAuthor%5D&cauthor=true&cauthor_uid=17537363), [Verdú J](https://www.ncbi.nlm.nih.gov/pubmed/?term=Verdú J%5BAuthor%5D&cauthor=true&cauthor_uid=17537363), [Sánchez S](https://www.ncbi.nlm.nih.gov/pubmed/?term=Sánchez S%5BAuthor%5D&cauthor=true&cauthor_uid=17537363), [Castaño V](https://www.ncbi.nlm.nih.gov/pubmed/?term=Castaño V%5BAuthor%5D&cauthor=true&cauthor_uid=17537363). Genetic thrombophilia and cerebral venous thrombosis. [*Med Clin (Barc).*](https://www.ncbi.nlm.nih.gov/pubmed/?term=Romero+2007+cerebral+thrombophliai) 2007;128:655-656.
36. [Altinisik J](https://www.ncbi.nlm.nih.gov/pubmed/?term=Altinisik J%5BAuthor%5D&cauthor=true&cauthor_uid=18160601), [Ates O](https://www.ncbi.nlm.nih.gov/pubmed/?term=Ates O%5BAuthor%5D&cauthor=true&cauthor_uid=18160601), [Ulutin T](https://www.ncbi.nlm.nih.gov/pubmed/?term=Ulutin T%5BAuthor%5D&cauthor=true&cauthor_uid=18160601), [Cengiz M](https://www.ncbi.nlm.nih.gov/pubmed/?term=Cengiz M%5BAuthor%5D&cauthor=true&cauthor_uid=18160601), [Buyru N](https://www.ncbi.nlm.nih.gov/pubmed/?term=Buyru N%5BAuthor%5D&cauthor=true&cauthor_uid=18160601). Factor V Leiden, prothrombin G20210A, and protein C mutation frequency in Turkish venous thrombosis patients. [*Clin Appl Thromb Hemost.*](https://www.ncbi.nlm.nih.gov/pubmed/18160601)2008;14:415-420.
37. [Koopman K](https://www.ncbi.nlm.nih.gov/pubmed/?term=Koopman K%5BAuthor%5D&cauthor=true&cauthor_uid=19187954), [Uyttenboogaart M](https://www.ncbi.nlm.nih.gov/pubmed/?term=Uyttenboogaart M%5BAuthor%5D&cauthor=true&cauthor_uid=19187954), [Hendriks HG](https://www.ncbi.nlm.nih.gov/pubmed/?term=Hendriks HG%5BAuthor%5D&cauthor=true&cauthor_uid=19187954), [Luijckx GJ](https://www.ncbi.nlm.nih.gov/pubmed/?term=Luijckx GJ%5BAuthor%5D&cauthor=true&cauthor_uid=19187954), [Cramwinckel IR](https://www.ncbi.nlm.nih.gov/pubmed/?term=Cramwinckel IR%5BAuthor%5D&cauthor=true&cauthor_uid=19187954), [Vroomen PC](https://www.ncbi.nlm.nih.gov/pubmed/?term=Vroomen PC%5BAuthor%5D&cauthor=true&cauthor_uid=19187954), et al. Thromboelastography in patients with cerebral venous thrombosis. [*Thromb Res.*](https://www.ncbi.nlm.nih.gov/pubmed/19187954) 2009;124:185-188.
38. [Laugesaar R](https://www.ncbi.nlm.nih.gov/pubmed/?term=Laugesaar R%5BAuthor%5D&cauthor=true&cauthor_uid=20337781), [Kahre T](https://www.ncbi.nlm.nih.gov/pubmed/?term=Kahre T%5BAuthor%5D&cauthor=true&cauthor_uid=20337781), [Kolk A](https://www.ncbi.nlm.nih.gov/pubmed/?term=Kolk A%5BAuthor%5D&cauthor=true&cauthor_uid=20337781), [Uustalu U](https://www.ncbi.nlm.nih.gov/pubmed/?term=Uustalu U%5BAuthor%5D&cauthor=true&cauthor_uid=20337781), [Kool P](https://www.ncbi.nlm.nih.gov/pubmed/?term=Kool P%5BAuthor%5D&cauthor=true&cauthor_uid=20337781), [Talvik T](https://www.ncbi.nlm.nih.gov/pubmed/?term=Talvik T%5BAuthor%5D&cauthor=true&cauthor_uid=20337781). Factor V Leiden and prothrombin 20210G>A [corrected] mutation and paediatric ischaemicstroke: a case-control study and two meta-analyses. [*Acta Paediatr.*](https://www.ncbi.nlm.nih.gov/pubmed/?term=Factor+V+Leiden+and+prothrombin+21210G>A+mutation+and+paediatric+ischaemic+stroke%3A+a+case–control+study+and+two+meta-analyses) 2010;99:1168-1174.
39. [Cesarman-Maus G](https://www.ncbi.nlm.nih.gov/pubmed/?term=Cesarman-Maus G%5BAuthor%5D&cauthor=true&cauthor_uid=21193750), [Cantú-Brito C](https://www.ncbi.nlm.nih.gov/pubmed/?term=Cantú-Brito C%5BAuthor%5D&cauthor=true&cauthor_uid=21193750), [Barinagarrementeria F](https://www.ncbi.nlm.nih.gov/pubmed/?term=Barinagarrementeria F%5BAuthor%5D&cauthor=true&cauthor_uid=21193750), [Villa R](https://www.ncbi.nlm.nih.gov/pubmed/?term=Villa R%5BAuthor%5D&cauthor=true&cauthor_uid=21193750), [Reyes E](https://www.ncbi.nlm.nih.gov/pubmed/?term=Reyes E%5BAuthor%5D&cauthor=true&cauthor_uid=21193750), [Sanchez-Guerrero J](https://www.ncbi.nlm.nih.gov/pubmed/?term=Sanchez-Guerrero J%5BAuthor%5D&cauthor=true&cauthor_uid=21193750), et al. Autoantibodies against the fibrinolytic receptor, annexin A2, in cerebral venous thrombosis. [*Stroke.*](https://www.ncbi.nlm.nih.gov/pubmed/?term=Autoantibodies+against+the+Fibrinolytic+Receptor%2C+Annexin+A2%2C+in+Cerebral+Venous+Thrombosis) 2011;42:501-503.
