# Supplementary material for: Prevalence and geographical variation of Factor V Leiden in patients with cerebral venous thrombosis: A meta-analysis
Source: PLoS One. 2018 Aug 29;13(8):e0203309. doi: 10.1371/journal.pone.0203309 (PMC6114929; doi:10.1371/journal.pone.0203309)
Supplement: S2 Table — (DOC) [file pone.0203309.s005.doc]

**S2 Table. Characteristics of 8 studies excluded from meta-analysis.**

| **Study** | **Year** | **Country** | **CVT ascertainment** | **CVT(N)** | **Male/ female (N)** | **Mean or median age(range), years** | **Controls (N)** | **Male/ female (N)** | **Age** | **Matched** |
| --- | --- | --- | --- | --- | --- | --- | --- | --- | --- | --- |
| **Martinelli** | 1996 | Italy | Intra-arterial angiography, intravenous DA,MRI or CT | 25 | 5/20 | 32 (21-64) | 75 | - | - | Age and sex |
| **Martinelli** | 1998 | Italy | CT, MRI or angiography | 40 | 9/31 | 31 (15-64) | 120 | 27/93 | 32 (18-64) | Age,sex,geographic origin and level of education |
| **Meng** | 2002 | China | Angiography | 20 | 8/12 | 31.0 (20-48) | 50 | 28/22 | 38.0 (18-58) | - |
| **Le Cam-Duchez** | 2005 | France | MRI or angiography | 26 | 8/18 | 40.9 (2-75) | 84 | 34/50 | 36.2 (20-62) | Age and sex |
| **Tufano** | 2005 | Italy | CT and/or MRI | 20 | 8/12 | 34.5(-) | 328 | 113/195 | 36.6(-) | Age and sex |
| **Dindagur** | 2007 | India | MRI/MRV | 50 | - | 23 (18-35) | 100 | - | 25 (18-40) | Age, the same geographic and socioeconomic group |
| **Lichy** | 2007 | Germany | Documented objectively | 76 | 17/59 | 37.0(-) | 195 | 87/108 | 38.5 | Same region |
| **Martinelli** | 2010 | Italy | Documented objectively | 107 | - | - | 842 | - | - | - |

CVT,cerebral venous thrombosis; CT;computed tomography; MRI,magnetic resonance imaging; MRV,magnetic resonance venography; DA,digital angiography; - ,not applicable.
